# Nitric Oxide Attenuates TGF-β-Induced Myofibroblast Differentiation Through Reversible Mitochondrial Bioenergetic Reprogramming Despite Compensatory Glycolytic Activation

**DOI:** 10.3390/cells15211929

**Published:** 2026-10-23

**Authors:** Madita Pflänzel, Juliana F. Bousch, Christoph V. Suschek, Carl Neuerburg, Lisa Oezel

**Affiliations:** Department for Orthopedics and Trauma Surgery, Medical Faculty and University Hospital Düsseldorf, Heinrich Heine University Düsseldorf, 40225 Düsseldorf, Germanyjulianafranziska.bousch@med.uni-duesseldorf.de (J.F.B.); carl.neuerburg@med.uni-duesseldorf.de (C.N.);

**Keywords:** myofibroblast differentiation, nitric oxide, alpha-smooth muscle actin (αSMA), glycolysis, mitochondrial respiration, ATP, cellular energy metabolism

## Abstract

Fibroproliferative disorders such as hypertrophic scars and sclerotic skin diseases are characterized by excessive fibroblast activation and differentiation into contractile myofibroblasts. Transforming growth factor-β (TGF-β) is a central driver of this process, promoting α-smooth muscle actin (α-SMA) expression, extracellular matrix production, and increased cellular energy demand. Nitric oxide (NO) is a key regulator of mitochondrial function and cellular bioenergetics and has been reported to exert both pro- and anti-fibrotic effects depending on the cellular context. However, the mechanisms linking NO-dependent metabolic regulation to fibroblast differentiation remain incompletely understood. Here, we demonstrate that exogenous NO, delivered by the long-acting donor DETA/NO, potently suppresses TGF-β-induced myofibroblast differentiation in primary human dermal fibroblasts. DETA/NO markedly reduced α-SMA, EDA-fibronectin, and collagen I expression while simultaneously inhibiting mitochondrial respiration, ATP-linked oxidative phosphorylation, and maximal respiratory capacity. Although NO elicited a pronounced compensatory increase in glycolytic ATP production, this response failed to preserve total cellular ATP availability, resulting in a substantial reduction in overall ATP production. Importantly, these metabolic alterations were fully reversible following removal of the NO donor, indicating transient metabolic regulation rather than irreversible cellular injury. Notably, enhanced glycolytic ATP production occurred despite profound inhibition of myofibroblast differentiation, demonstrating that increased glycolytic ATP production alone is insufficient to sustain the fibrogenic phenotype when cellular ATP availability is compromised. Inhibition of soluble guanylate cyclase by ODQ failed to reverse the anti-differentiative effects of NO, indicating that canonical NO–cGMP signaling is not required for this response. Conversely, inhibition of mitochondrial ATP synthesis by oligomycin phenocopied both the metabolic and anti-differentiative effects of NO. Collectively, our findings identify NO as a reversible regulator of mitochondrial bioenergetics and fibroblast activation and demonstrate that maintenance of cellular ATP homeostasis, rather than enhanced glycolytic ATP production per se, is a critical metabolic requirement for efficient myofibroblast differentiation. Targeting cellular bioenergetics may therefore represent a promising strategy for preventing pathological fibrosis and hypertrophic scar formation.

## 1. Introduction

Fibroproliferative and sclerotic skin disorders, including hypertrophic scars, keloids, and Dupuytren’s contracture, represent significant clinical challenges due to dysregulated tissue remodeling and persistent fibrotic activation. Although their etiologies differ, these conditions share a common pathogenic hallmark: excessive fibroblast activation and differentiation into contractile myofibroblasts. This process is driven by elevated levels of growth factors, cytokines, and profibrotic mediators that promote extracellular matrix deposition and progressive tissue stiffening [1,2].

Myofibroblasts arise from resident dermal fibroblasts, mesenchymal progenitors, epithelial cells via epithelial–mesenchymal transition, or bone marrow-derived cells [3]. They are characterized by the expression of α-smooth muscle actin (α-SMA) incorporated into stress fibers, conferring enhanced contractility and matrix-remodeling capacity [2,4]. During physiological wound repair, fibroblast-to-myofibroblast differentiation contributes to wound contraction and subsequent tissue remodeling, with TGF-β1 signaling playing a central role in the acquisition of the contractile phenotype [5,6,7]. During later stages of tissue repair, extracellular matrix organization is progressively remodeled, cellularity declines, and scar tensile strength increases [8,9,10]. Appropriate resolution of the myofibroblast response is therefore essential for restoration of tissue homeostasis, whereas dysregulation of this process may result in either insufficient repair or excessive scar formation [11]. Persistent myofibroblast activation, together with excessive extracellular matrix and collagen deposition, is a characteristic feature of fibroproliferative disorders such as hypertrophic scars and keloids [8,9,10,12,13]. These lesions are further associated with impaired removal of activated myofibroblasts by apoptosis [2,14,15], emphasizing the central role of persistent myofibroblast activity in pathological tissue remodeling [5,16]. TGF-β is widely recognized as a major mediator of fibroblast-to-myofibroblast differentiation, promoting α-SMA expression, collagen synthesis, proliferation, and resistance to apoptosis [17].

Current therapeutic approaches for fibrotic skin conditions, including corticosteroids, immunosuppressants, laser therapy, cryotherapy, radiation, and surgical excision, are often associated with high recurrence rates and limited long-term efficacy [18,19,20,21,22,23]. Although silicone-based applications and mechanotherapeutic strategies offer partial benefit [24,25], effective molecularly targeted interventions remain scarce. Emerging antifibrotic strategies focus on inflammatory signaling, epithelial–mesenchymal transition, and direct inhibition of TGF-β signaling pathways [26,27,28].

Increasing evidence indicates that fibroblast differentiation is tightly coupled to cellular metabolism. TGF-β-driven myofibroblast activation requires substantial energetic support, accompanied by metabolic reprogramming that includes enhanced glycolysis and mitochondrial oxidative phosphorylation [29,30,31,32]. Glycolytic upregulation was first noted in studies of cystic fibrosis [32,33,34] and later confirmed in various fibroblast populations [35,36]. Upregulation of glycolysis-associated genes, many of which serve as metabolic checkpoints, further facilitates this transition [35,37,38]. Glycolysis inhibitors like 2-deoxyglucose (2DG) have been shown to block α-SMA expression, leading to cell cycle arrest, diminished ATP synthesis, and reduced cell viability [35,38,39,40].

In line with this concept, we previously demonstrated that chemically or physically mediated modulation of cellular energy metabolism significantly attenuated fibroblast differentiation. Thus, exposure to CO_2_-saturated solutions [41] or blue light at l = 453 nm [42] effectively reduced TGF-β-induced fibroblast differentiation and correlated with a reversible, nearly completely suppressed mitochondrial respiration, significantly lowered glycolytic flux, and strongly decreased ATP synthesis without inducing cytotoxicity. These findings suggested that transient interference with cellular bioenergetics may represent a viable antifibrotic strategy.

Nitric oxide (NO) is a pleiotropic gaseous signaling molecule involved in inflammation, vascular tone regulation, and redox homeostasis [43,44]. Beyond its classical signaling functions, NO is known to modulate cellular metabolism [45,46,47]. It can reversibly inhibit components of the mitochondrial electron transport chain, alter redox balance, and induce post-translational modifications such as S-nitrosylation of metabolic enzymes, including glyceraldehyde-3-phosphate dehydrogenase (GAPDH) [48,49,50]. Through these mechanisms, NO has the capacity to influence both oxidative phosphorylation and glycolysis. Despite extensive characterization of NO in vascular and immune systems, its impact on the metabolic control of human skin fibroblast differentiation has not been systematically investigated. Given the central role of glycolytic or mitochondrial ATP production in TGF-β-induced fibroblast differentiation, we hypothesized that exogenously applied NO may interfere with myofibroblast differentiation through modulation of cellular bioenergetics.

In the present study, we examined the effects of the long-acting NO donor DETA/NO on TGF-β-induced differentiation of primary human dermal fibroblasts, and found that exogenously applied NO significantly attenuates spontaneous and TGF-β-induced myofibroblast formation. This effect was accompanied by a reversible suppression of mitochondrial respiration, a compensatory shift toward glycolysis, and a net reduction in total ATP production. Our findings identify nitric oxide as a reversible metabolic regulator of fibroblast differentiation and suggest that transient modulation of cellular bioenergetics may represent a novel approach to mitigate pathological skin fibrosis and scar formation.

## 2. Materials and Methods

### 2.1. Materials

Unless otherwise stated, all chemicals, antibodies, and assay kits were sourced from Sigma-Aldrich Chemie GmbH (Munich, Germany).

### 2.2. Nitric Oxide Donor

The NO donor (Z)-1-[N-(2-aminoethyl)-N-(2-ammonioethyl)ami-no]diazen-1-ium-1,2-diolate (DETA/NO) was kindly provided by Prof. K. D. Kröncke (Heinrich-Heine-University of Düsseldorf, Düsseldorf, Germany). Denitrosilated DETA/NO (DETA) was obtained by incubating stock solutions (50 mM) of DETA/NO for 10 days at 37 °C. DETA/NO spontaneously releases NO with a predictable first-order kinetic and with a half-life of 7.7 h, thus providing a constant NO supply over hours [51,52]. According to the first-order kinetic laws (t_1/2_ = ln2/k and c = c_0_ × e^−kt^), 100 µM of DETA/NO generates 112 nM NO per minute.

### 2.3. Cell Culture

Primary human dermal fibroblasts were isolated from skin samples obtained with written informed consent from twelve patients (eight female, four male; age range 42–63 years) undergoing elective breast reduction or abdominoplasty procedures, respectively. Tissue procurement and cell isolation were performed under standardized laboratory conditions as previously established in our laboratory [20].

Immediately after surgical excision, skin specimens were transferred to sterile culture dishes and mechanically minced into small fragments. The tissue was incubated overnight at 4 °C in Dispase II to facilitate separation of epidermal and dermal layers. On the following day, the epidermis was carefully removed, and the remaining dermal compartment was subjected to enzymatic digestion using collagenase at 37 °C for one hour under continuous agitation.

Following enzymatic dissociation, the cell suspension was gently triturated and passed through a sterile mesh filter (50–100 µm pore size) to eliminate residual tissue debris. The filtrate was centrifuged, and the resulting cell pellet was resuspended in Dulbecco’s Modified Eagle Medium (DMEM) containing 4.5 g/l glucose. Cells were then seeded into culture flasks and maintained at 37 °C in a humidified incubator with 5% CO_2_ until further expansion and experimentation. Fibroblasts (FB) were used for experiments in passages 2–6.

All procedures involving human tissue were approved by the Ethics Committee of the Medical Faculty of Heinrich Heine University Düsseldorf (approval no. 3634) and conducted in accordance with the principles outlined in the Declaration of Helsinki.

### 2.4. Experimental Procedures

Primary human dermal fibroblasts (FB; passages 2–6) were seeded at defined densities onto standard tissue culture plates (96-well for viability assays, 96-well XF24 cell culture microplates for Seahorse analysis, 24-well for PCR, 6-well for Western blot) and maintained in Dulbecco’s Modified Eagle Medium supplemented with 10% fetal calf serum (FCS) until reaching 80–90% confluency. Cells were cultured at 37 °C in a humidified incubator with 5% CO_2_ under normoxic conditions.

To induce myofibroblast differentiation, FB were stimulated with transforming growth factor-β (TGF-β; 2 ng/mL) for 72 h. Where indicated, cultures were treated in parallel with the nitric oxide donor DETA/NO at concentrations of 100 µM or 300 µM, Oligomycin or the soluble guanylate cyclase inhibitor ODQ at concentrations indicated, or as a control with decomposed DETA/NO solutions containing equimolar amounts of the carrier molecule DETA and nitrite (NO_2_), respectively. The medium, along with all supplements, was replaced after 24 and 48 h. Untreated cultures served as baseline controls. After the 72-h incubation period, cells were harvested and lysed for protein extraction. Protein expression levels of differentiation-associated markers (aSMA, EDA-Fibronectin) were analyzed by Western blotting techniques. In addition, mRNA expression levels were quantified by real-time PCR to assess transcriptional regulation under the respective experimental conditions.

For assessment of cell viability and proliferation, fibroblasts were seeded at a density of 500 cells per well in 96-well plates and cultured under the respective experimental conditions for a period of four days. The medium, along with all supplements, was replaced after 24 and 48 h. Cell growth was quantified by microscopy-based analysis of nuclear counts after chromatin staining with Hoechst 33342 dye following imaging of the cultures. Quantification was performed by automated counting of stained nuclei using standardized imaging parameters. Metabolic viability assays such as MTT or resazurin-based (CellTiter-Blue) assays were deliberately not employed. Since nitric oxide directly interferes with mitochondrial respiration and cellular redox metabolism, these assays may produce artifactual results due to their dependence on mitochondrial enzymatic activity and intracellular reduction potential. To avoid confounding effects related to NO-induced modulation of oxidative phosphorylation and redox state, we therefore chose direct microscopic cell counting as a metabolically independent and unbiased method to assess cell proliferation and viability. Independent biological replicates (*n*) ranged from *n* = 3 to *n* = 6 (as specified in the respective figure legends).

### 2.5. Immunocytochemistry

Assessment of myofibroblast differentiation was performed by immunocytochemical detection of α-smooth muscle actin (α-SMA) and Collagen I. Fibroblasts were fixed with 4% paraformaldehyde for 15 min at ambient temperature to preserve cellular architecture. Membrane permeabilization was subsequently achieved using 0.2% Triton X-100. To minimize nonspecific antibody binding, cells were incubated for 30 min in a blocking solution consisting of phosphate-buffered saline (PBS) supplemented with 4% bovine serum albumin (BSA; PAA, Colbe, Germany).

Primary antibody incubation was carried out for 60 min using a monoclonal mouse anti-human α-SMA (ab7817, Abcam, Cambridge, UK) or monoclonal mouse anti-human collagen I (ab6308, Abcam) diluted 1:400 in blocking buffer. Following incubation, cells were rinsed three times with PBS to remove residual antibody. Signal detection was performed using the Dako visualization system (DakoCytomation, Hamburg, Germany) according to the manufacturer’s protocol. Representative images from each well were captured to assess the degree of myofibroblast differentiation [53].

### 2.6. Real-Time qPCR

For real-time qPCR, total cell RNA was isolated after lysing fibroblasts, maintained in 6-well plates, using the RNeasy^®^ Mini Kits (QIAGEN N.V., Hilden, Germany). The RNA was converted into cDNA using the Omniscript^®^ RT Kits (QIAGEN N.V., Hilden, Germany). The RT-qPCR was performed in triplicate using the Power SYBR^®^ Green PCR Master Mix (Thermo Fisher Scientific GmbH, Karlsruhe, Germany) with 1 ng cDNA for aSMA (Forward Primer (5′->3′): AGCCAAGCACTGTCAGGAAT; Reverse Primer (5′->3′): TTGTCACACACCAAGGCAGT; Product Length: 108 bp) in a 25 µL PCR reaction with a 7300 Real-Time PCR System (Applied Biosystems, Waltham, MA, USA). For the determination of mRNA expression, primer concentrations of 0.2 µM were used. Transferrin receptor 1 (TFRC) was used as the reference gene for normalization (Forward Primer (5′->3′): TTCAGGTCAAAGACAGCGCTCA; Reverse Primer (5′->3′): CTATACGCCACATAACCCCC-AGG; Product Length: 100 bp). The 2^−ΔΔCt^ method was employed for the calculation of relative mRNA expression.

### 2.7. Cell Protein Collection, Gel Electrophoresis and Western Blotting

For protein extraction, fibroblast cultures were first rinsed with ice-cold phosphate-buffered saline (PBS, 4 °C) to remove residual medium. Cells were subsequently lysed directly in culture dishes using 40 µL RIPA lysis buffer supplemented with protease inhibitors (7× stock solution; Roche, Basel, Switzerland; final concentration 14.2%). The lysates were subjected to brief sonication to ensure complete protein solubilization, followed by centrifugation at 20,000× *g* for 10 min. The supernatants containing soluble proteins were collected for further analysis.

Protein concentrations were determined using the Pierce™ BCA Protein Assay Kit (#23225, Thermo Fisher, Dreieich, Germany) according to the manufacturer’s instructions. Equal amounts of total protein (30 µg per lane) were separated by SDS-PAGE under reducing conditions using the Xcell SureLock™ Mini-Cell system (Invitrogen, Karlsruhe, Germany). Proteins were then transferred onto nitrocellulose membranes (Peqlab, Erlangen, Germany).

Membranes were incubated overnight at 4 °C in TBS-T buffer (T-TBS) containing 5% non-fat dry milk and 0.1% Tween 20 to block nonspecific binding sites. Primary rabbit anti-human antibodies directed against EDA-fibronectin (ab6328, Abcam, Cambridge, UK) and α-smooth muscle actin (ab7817, Abcam) were applied at dilutions recommended by the manufacturer.

After three washing steps (5 min each in T-TBS), membranes were incubated for 1 h with horseradish peroxidase–conjugated goat anti-rabbit IgG/HRP (#P0449; Agilent Technologies, Santa Clara, CA, USA) secondary antibody (1:1000 dilution). Following four additional washing steps (3 min each in T-TBS), immunoreactive bands were visualized using the Clarity™ Western ECL detection reagent (BioRad, #170-5060). Signal intensities were quantified densitometrically with Quantity One 1-D analysis software (version 4.6.5; BioRad). The value of the protein expression of interest was normalized to the value of the corresponding signal of the total amount of proteins of the respective samples detected on the nitrocellulose membrane.

### 2.8. cGMP-Measurement

Nitric oxide-induced activation of soluble guanylate cyclase was assessed by measuring intracellular cGMP accumulation in NO-sensitive RFL-6 reporter cells as previously described [54]. RFL-6 cells were seeded into six-well plates at a density of 3 × 10^5^ cells per well and grown to confluence under standard culture conditions. To prevent degradation of newly synthesized cGMP and to preserve NO bioavailability, cells were incubated in medium containing superoxide dismutase (SOD, 500 U/mL) and the phosphodiesterase inhibitor isobutylmethylxanthine (IBMX, 0.6 mM).

For stimulation experiments, confluent cells were treated with 300 µM DETA/NO either in the absence or presence of the selective soluble guanylate cyclase inhibitor ODQ (30 µM). After incubation for 2 h, cells were mechanically detached and lysed by repeated freeze–thaw cycles. Intracellular cGMP levels were quantified using a competitive enzyme immunoassay (Cayman Chemical, Ann Arbor, MI, USA) according to the manufacturer’s protocol. Measured cGMP concentrations were normalized to total cellular protein content and expressed as nmol cGMP per mg protein.

### 2.9. Cell Preparation for Seahorse^®^ Assay

Mitochondrial respiration was analyzed by determining the oxygen consumption rate (OCR) using the Agilent Seahorse XF24/XFe24 Extracellular Flux Analyzer (Seahorse Bioscience, North Billerica, MA, USA) in combination with the standardized Mito Stress Test protocol [55,56]. For these measurements, adherent 2 × 10^4^ fibroblasts were seeded into XF24 cell culture microplates and allowed to attach overnight. The treatment with DETA/NO was carried out overnight for a period of 8 h immediately prior to measurement. In the experiments investigating the reversibility of the NO effect, the cell cultures pre-incubated with DETA/NO for 8 h were washed twice, cultivated in normal medium for 24 h, and subsequently subjected to Seahorse analysis.

Prior to analysis, the culture medium was replaced with Seahorse XF base medium (pH 7.4) lacking bicarbonate and CO_2_ buffering, supplemented with 10 mM glucose, 2 mM glutamine, and 1 mM pyruvate. Plates were equilibrated for one hour at 37 °C in a non-CO_2_ incubator.

Mitochondrial function was assessed through sequential injections via the sensor cartridge ports. Oligomycin (1–2 µM) was first applied to inhibit ATP synthase (Complex V) and determine ATP-linked respiration. Subsequently, FCCP (carbonyl cyanide 4-(trifluoromethoxy) phenylhydrazone; 0.5–1 µM) was added to uncouple oxidative phosphorylation and assess maximal respiratory capacity. Finally, rotenone (Complex I inhibitor) and antimycin A (Complex III inhibitor; 0.5 µM each) were injected to completely block mitochondrial respiration, allowing calculation of non-mitochondrial oxygen consumption. Each measurement cycle consisted of mixing, equilibration, and recording phases of approximately five minutes and was repeated three to five times per condition. OCR values were expressed as pmol O_2_ per minute.

In parallel, glycolytic activity was evaluated by measuring the extracellular acidification rate (ECAR), which serves as a surrogate marker for glycolytic flux [57]. After equilibration in assay medium, basal ECAR was recorded, followed by sequential injections of glucose (10 mM) to initiate glycolysis, oligomycin to induce maximal glycolytic capacity via mitochondrial inhibition, and 2-deoxyglucose (2-DG; 50 mM) to block glycolysis and determine non-glycolytic acidification. These steps enabled calculation of basal glycolysis, glycolytic capacity, and glycolytic reserve.

To further delineate the relative contributions of oxidative phosphorylation and glycolysis to cellular ATP production, we employed the Seahorse XF ATP Rate Assay [58]. Cells were prepared and equilibrated as described for the mitochondrial stress assay. During measurement, cells were maintained in XF assay medium supplemented with 10 mM glucose, 2 mM glutamine, and 1 mM pyruvate (pH 7.4). Oligomycin (1.5 µM) was injected to inhibit ATP synthase and determine glycolytic ATP production, followed by combined rotenone and antimycin A (0.5 µM each) to terminate mitochondrial respiration and quantify non-mitochondrial oxygen consumption. Using Seahorse Wave software(Report Generator, Version: 4.03) and the integrated ATP Rate calculation module, OCR and ECAR values were converted into absolute mitochondrial and glycolytic ATP production rates (pmol ATP/min). Both absolute ATP production and relative pathway contributions were analyzed for untreated and treated cells, including NO-exposed conditions. Data normalization was performed based on protein concentration or cell number per well.

All metabolic measurements were performed under experimental conditions consistent with those previously established in our laboratory for primary human skin fibroblast cell cultures [41,42]. After completion of each assay, data were normalized to total protein content or cell number per well. Metabolic parameters were calculated using Seahorse Wave software and the corresponding Mito Stress Test report generator.

### 2.10. Statistical Analysis

Statistical analyses were performed using GraphPad Prism 8 (GraphPad Software, San Diego, CA, USA). Comparisons involving multiple experimental conditions were performed using one-way ANOVA followed by Tukey’s post hoc multiple-comparison test. For comparisons between two groups, the non-parametric Wilcoxon test was used. Data are presented as mean ± SD. The number of independent biological replicates (*n*) is indicated in the corresponding figure legends. A *p*-value < 0.05 was considered statistically significant.

## 3. Results

### 3.1. Effects of Nitric Oxide on TGF-β-Induced Myofibroblast Differentiation of Fibroblasts

**Figure 1:**
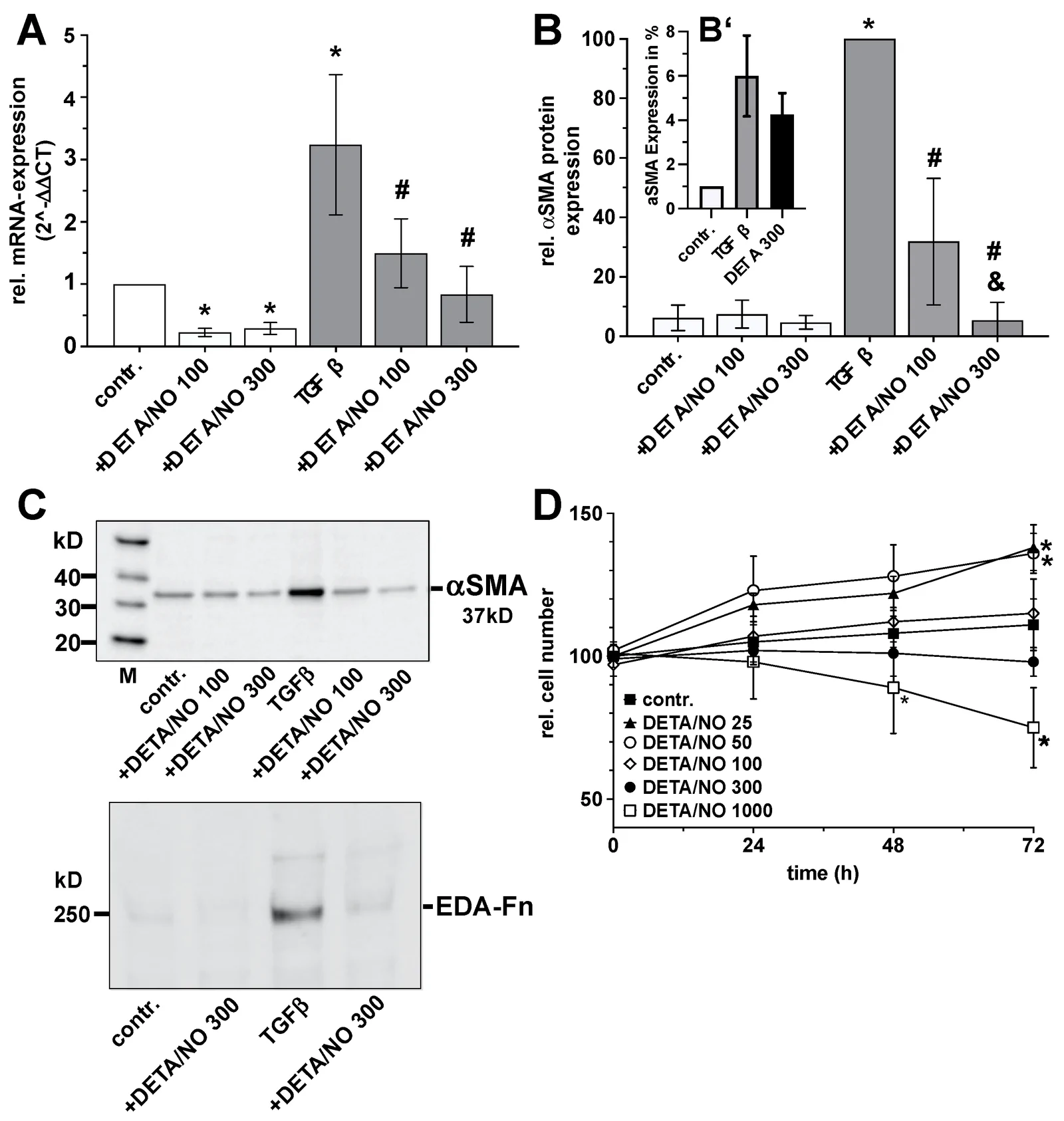
**Effects of nitric oxide on TGF-β-induced myofibroblast differentiation of primary human skin fibroblasts.** Untreated or TGF-β-activated (2 ng/mL) primary human skin fibroblast cultures were maintained for 72 h in the presence or absence of the NO-donor DETA/NO (100 or 300 µM). After cell lysis and mRNA isolation, α-SMA mRNA expression was quantified by rtPCR (**A**), and after protein processing, the expression of α-SMA (**B**,**C**) and EDA-Fibronectin (**C**) was quantified by Western blot technique. (**B’**), As a control for the effect specificity of nitric oxide, we used the NO-free donor after decomposition (three independent biological replicates; *n* = 3). (**A**,**B**), White bars, Untreated cell cultures; gray bars, TGF-β-activated fibroblast cultures; black-white striped bar in (**B’**), TGF-β-activated fibroblast cultures treated with decomposed DETA/NO (300 µM DETA). Values represent the mean ± S.D. of six individual experiments (six independent biological replicates; *n* = 6). *, *p* < 0.05 as compared to the control (contr.); ^#^, *p* < 0.05, as compared to only with TGF-β-activated fibroblast cultures; ^&^, *p* < 0.05 as compared to TGF-β-activated fibroblast cultures incubated with 100 µM DETA/NO. (**C**), Shown are representative images of α-SMA and EDA-Fibronectin protein expression from independent experiments (six independent biological replicates; *n* = 6) after Western blot analysis. (**D**), A 96-well cell culture plate confluent with fibroblasts was incubated with the indicated concentrations of DETA/NO (25–1000 µM) for a period of 72 h. At the beginning and every 24 h thereafter, cell number was quantified by counting nuclei stained with the dye Hoechst 33342. Values represent the mean ± S.D. of four individual experiments (four independent biological replicates; *n* = 4). *, *p* < 0.05 as compared with the control cultures maintained in the absence of DETA/NO (contr.).

TGF-β significantly increased α-SMA mRNA (Figure 1A) as well as protein expression levels (Figure 1B,C) and, in addition, EDA-Fibronectin protein expression, both confirming effective induction of myofibroblast differentiation. Treatment with the nitric oxide donor DETA/NO resulted in a significant reduction in TGF-β-induced α-SMA mRNA and protein expression, whereas a stronger reduction was observed with 300 µM DETA/NO as compared to results obtained with 100 µM DETA/NO (Figure 1A–C). Additionally, 300 µM DETA/NO significantly reduced TGF-β-induced EDA-Fibronectin protein expression Figure 1C. With the decomposed NO-donor, the mother substance alone (DETA) did not exert any significant effect on TGF-β-induced α-SMA protein expression (Figure 1B’). These results suggest an NO-specific mechanism, a claim that we were further able to support with the results of the viability assays (Figure 1D). In these assays, it was clearly evident that DETA/NO exerted a concentration-dependent biphasic effect on fibroblast growth. Low concentrations (25 and 50 µM) resulted in a modest but statistically significant increase in cell growth, whereas marked growth inhibition was observed only at the highest concentration tested (1000 µM). Importantly, at the concentrations used in the subsequent differentiation experiments (100 and 300 µM), DETA/NO did not significantly affect fibroblast growth. Thus, the inhibition of myofibroblast differentiation observed at 100 and 300 µM cannot be attributed to a general suppression of fibroblast growth, but rather supports a specific effect of DETA/NO on the differentiation process (Figure 1D).

**Figure 2:**
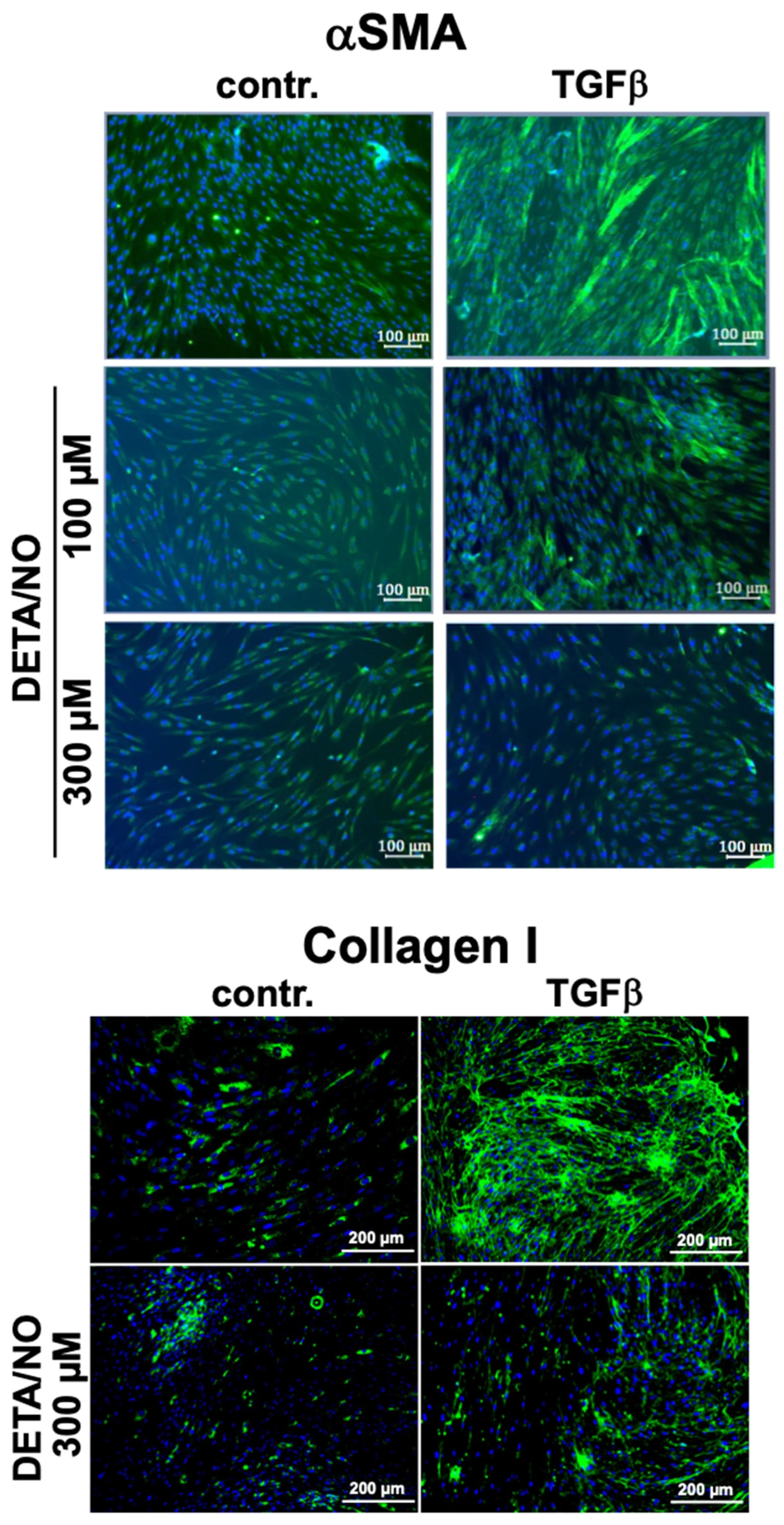
**Effects of nitric oxide on TGF-β-induced α-SMA and collagen I expression in primary human dermal fibroblasts.** Representative immunocytochemical images of α-SMA and collagen I expression in untreated control fibroblasts and fibroblasts stimulated with TGF-β (2 ng/mL) for 72 h in the absence or presence of DETA/NO. For α-SMA analysis, cells were treated with 100 or 300 µM DETA/NO; for collagen I analysis, cells were treated with 300 µM DETA/NO. α-SMA and collagen I immunoreactivity are shown in green, and cell nuclei were counterstained with Hoechst 33342 (blue). Images are representative of independent experiments (four independent biological replicates; *n* = 4). Scale bars: 100 µm for α-SMA and 200 µm for collagen I.

Furthermore, immunocytochemical analyses confirmed the differentiation-inhibiting effect of nitric oxide at the cellular level (Figure 2). Using α-SMA-specific or Collagen I-specific fluorescence-labeled antibodies, we observed the expected strong increase in the α-SMA- or Collagen I-specific green fluorescence signal following incubation with TGF-β. These signals were markedly reduced in cultures treated with 100 µM DETA/NO and almost completely diminished in cell cultures treated with 300 µM DETA/NO (Figure 2).

### 3.2. Nitric Oxide Reversibly Suppresses Mitochondrial Respiration in Human Dermal Skin Fibroblasts

**Figure 3:**
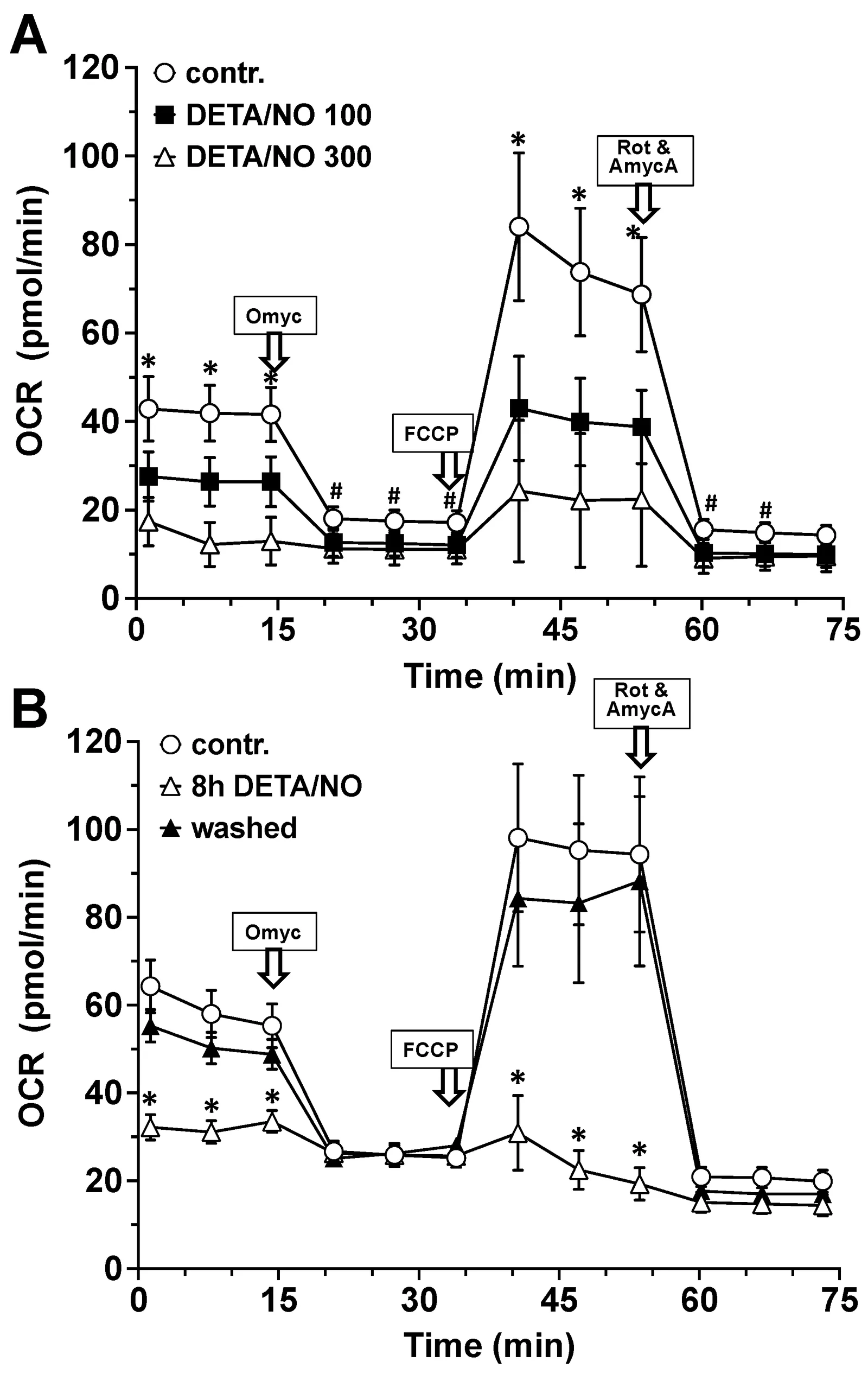
**Nitric oxide reversibly suppresses mitochondrial respiration in human dermal skin fibroblasts.** Impact on nitric oxide (8 h preincubation with DETA/NO 100 µM and 300 µM prior analysis) on mitochondrial function of human skin fibroblasts analyzed using the Seahorse XF Mito Stress Test, allowing real-time measurement of oxygen consumption rate (OCR) under basal conditions and after sequential injection of oligomycin (Omyc, reflecting ATP-linked respiration), FCCP (indicating preserved maximal respiratory capacity), and rotenone/antimycin A (Rot & AmycA). (**A**), Open circles, control cultures (contr.); black squares, 100 µM DETA/NO, white triangles, 300 µM DETA/NO. Values represent the mean ± S.D. of five individual experiments (five independent biological replicates; *n* = 5). *, *p* < 0.05 as compared to fibroblast cultures treated with 100 (black squares) or 300 µM DETA/NO (white triangles); ^#^, *p* < 0.05, as compared to fibroblast cultures treated with 300 µM DETA/NO (white triangles). (**B**), Open circles, control cultures (contr.); white triangles, 8 h incubation with 300 µM DETA/NO; black triangles, 8 h incubation with 300 µM DETA/NO prior 2 washing steps and 24 h incubation in medium. Values represent the mean ± S.D. of five individual experiments (five independent biological replicates; *n* = 5). *, *p* < 0.05 as compared to control fibroblast cultures (open circles) or fibroblast cultures treated for 8 h with 300 µM DETA/NO (black triangles).

Mitochondrial function was analyzed using the Seahorse XF Mito Stress Test, allowing real-time measurement of oxygen consumption rate (OCR) under basal conditions and after sequential injection of oligomycin, FCCP, and rotenone/antimycin A (Figure 3).

Under basal conditions, untreated control fibroblasts exhibited stable oxygen consumption rates with a clear response to mitochondrial modulators (Figure 3). Oligomycin injection resulted in the expected decrease in OCR, reflecting ATP-linked respiration, followed by a marked increase upon FCCP administration, indicating preserved maximal respiratory capacity. In contrast, fibroblasts treated with DETA/NO for 8 h showed a significant concentration-dependent reduction in basal OCR compared to untreated controls. The reduction was evident prior to oligomycin injection and persisted throughout the assay. ATP-linked respiration was markedly diminished, and FCCP-induced maximal respiration was significantly blunted, demonstrating that DETA/NO impairs both basal and maximal mitochondrial respiratory capacity (Figure 3A).

To assess whether this inhibition was reversible, fibroblasts were exposed to DETA/NO for 8 h, followed by washing and incubation in fresh medium for an additional 24 h before Seahorse analysis (Figure 3B). Strikingly, OCR measurements performed after the recovery period demonstrated substantial restoration of mitochondrial function. Basal respiration, ATP-linked respiration, and FCCP-induced maximal respiratory capacity were largely comparable to untreated controls. These findings demonstrate that DETA/NO-induced mitochondrial inhibition is not permanent but reversible upon removal of the NO donor (Figure 3B).

### 3.3. Nitric Oxide Reversibly Modulates Glycolytic Activity in Human Dermal Fibroblasts

**Figure 4:**
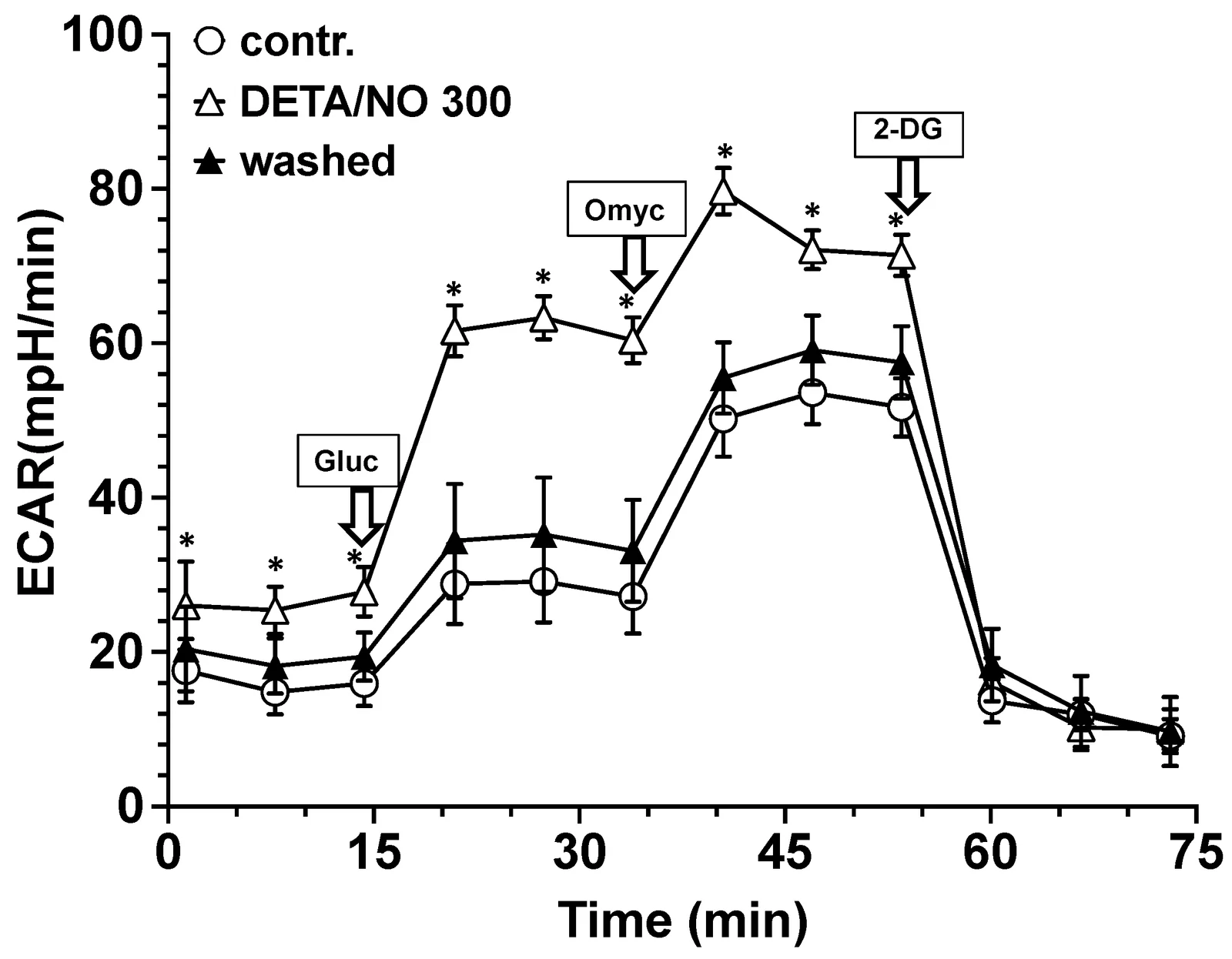
**Nitric oxide reversibly modulates glycolytic activity in human dermal fibroblasts.** To assess the impact of nitric oxide on glycolytic metabolism, extracellular acidification rate (ECAR) was measured using the Seahorse Glycolysis Stress Test. The assay included sequential injections of glucose (Gluc, reflecting activation of glycolysis), oligomycin (Omyc, indicating compensatory upregulation of glycolysis in response to inhibition of mitochondrial ATP synthesis), and 2-deoxyglucose (2-DG), enabling determination of basal glycolysis, glycolytic capacity, and glycolytic reserve. Open circles represent control cultures (contr.); white triangles represent cultures incubated for 8 h with 300 µM DETA/NO prior to measurement; black triangles represent cultures incubated for 8 h with 300 µM DETA/NO prior to 2 washing steps and 24 h incubation in medium. Values represent the mean ± S.D. of five individual experiments (five independent biological replicates; *n* = 5). *, *p* < 0.05 as compared with control fibroblast cultures (open circles) or DETA/NO-treated and washed fibroblast cultures (black triangles).

To assess the impact of nitric oxide on glycolytic metabolism, extracellular acidification rate (ECAR) was measured using the Seahorse Glycolysis Stress Test (Figure 4). The assay included sequential injections of glucose, oligomycin (Omyc), and 2-deoxyglucose (2-DG), enabling determination of basal glycolysis, glycolytic capacity, and glycolytic reserve.

Under control conditions, fibroblasts displayed the expected ECAR response pattern: Following glucose injection, ECAR increased markedly, reflecting activation of glycolysis (Figure 4). Subsequent addition of oligomycin further elevated ECAR, indicating compensatory upregulation of glycolysis in response to inhibition of mitochondrial ATP synthesis. Finally, 2-DG injection suppressed ECAR to near-baseline levels, confirming that the measured acidification was glycolysis-dependent. In contrast, fibroblasts treated with 300 µM DETA/NO exhibited a clearly altered glycolytic response. Basal ECAR after glucose injection was significantly enhanced as compared to untreated controls, representing a significantly higher basal glycolytic activity. Furthermore, oligomycin-induced maximal glycolytic capacity was also significantly increased. The glycolytic reserve was therefore strongly enhanced under DETA/NO exposure (Figure 4).

To determine whether this enhancement in metabolic glycolysis was reversible, fibroblasts were again treated with DETA/NO for 8 h, washed extensively, and incubated for an additional 24 h prior to ECAR analysis. After this recovery period, glycolytic parameters were largely restored. Basal glycolysis, glycolytic capacity, and glycolytic reserve were comparable to untreated control cells (Figure 4). The characteristic ECAR response to glucose, oligomycin, and 2-DG reappeared, indicating functional recovery of glycolytic metabolism, suggesting that DETA/NO-induced metabolic modulation is transient and reversible.

### 3.4. Nitric Oxide Shifts ATP Production from Mitochondrial Oxidative Phosphorylation Toward Glycolysis Independently of TGF-β Activation

**Figure 5:**
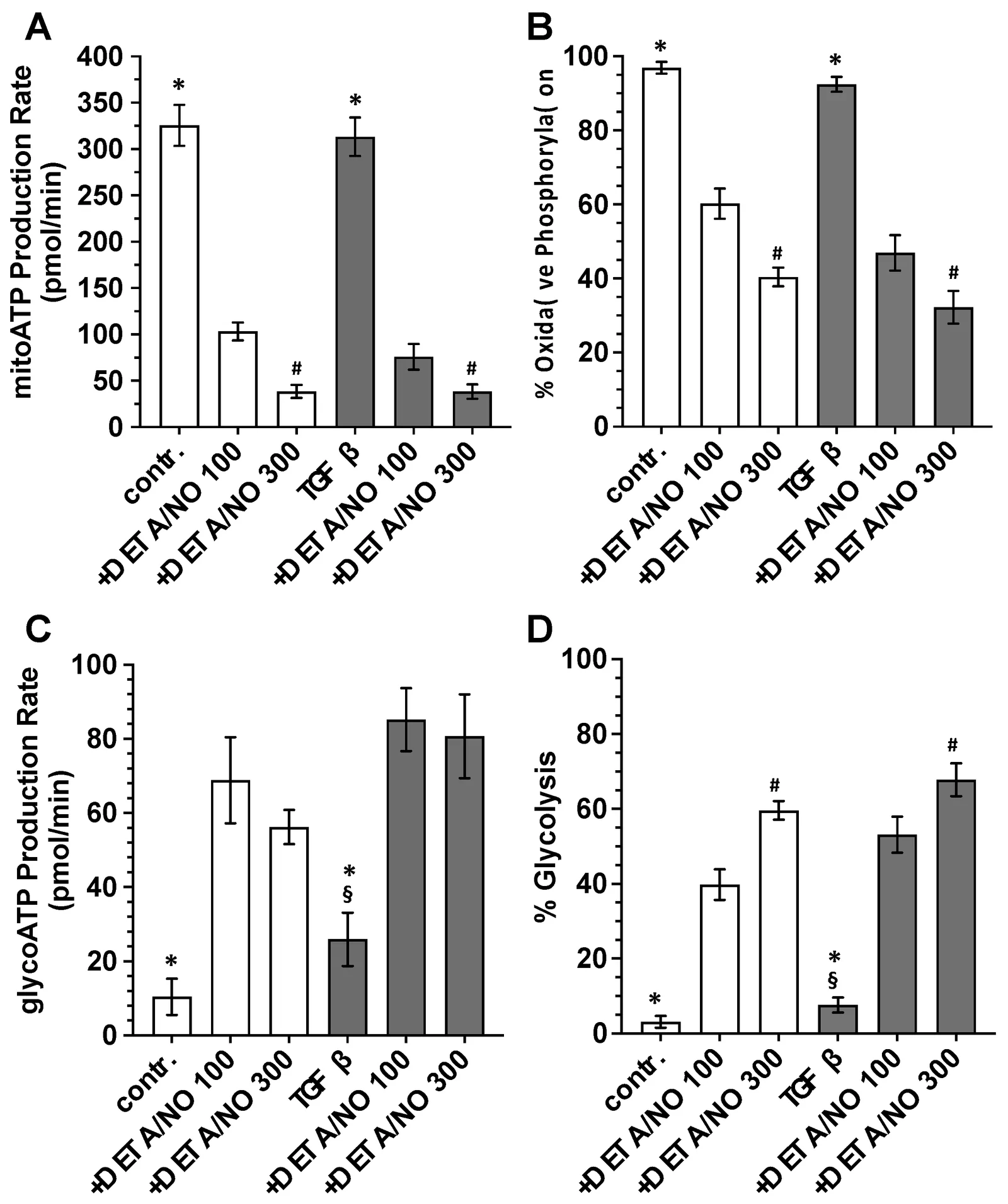
**Impact of nitric oxide on ATP production from mitochondrial oxidative phosphorylation or glycolysis.** Untreated or TGF-β-activated (2 ng/mL) primary human skin fibroblast cultures were maintained for 72 h in the presence or absence of the NO-donor DETA/NO (100 or 300 µM). Using the Seahorse XF ATP Rate Assay, absolute and relative parameters for mitochondrial and glycolytic ATP synthesis were recorded in parallel. (**A**), Mitochondrial ATP production rates (pmol/min); (**B**), Percentage contribution of oxidative phosphorylation to total ATP production; (**C**), glycolytic ATP production rates (pmol/min); (**D**), Percentage contribution of glycolysis to total ATP production. White bars, untreated cell cultures; gray bars, TGF-β-activated fibroblast cultures; Values represent the mean ± S.D. of five individual experiments (five independent biological replicates; *n* = 5). *, *p* < 0.05 as compared to the respective DETA/NO-treated cultures; ^#^, *p* < 0.05 as compared to the respective cultures incubated with 100 µM DETA/NO; ^§^, *p* < 0.05 as compared to non-activated control cultures (contr.).

ATP production rates were measured using the Seahorse XF ATP Rate Assay, allowing differentiation between mitochondrial and glycolytic ATP synthesis (Figure 5).

In untreated fibroblast cultures, mitochondrial ATP production (Figure 5A,B) clearly exceeded glycolytic ATP generation (Figure 5C,D), accounting for approximately 95% of total ATP production (Figure 5B). Upon treatment with the NO donor DETA/NO, a marked metabolic shift was observed: glycolytic ATP production significantly increased (Figure 5C,D) while mitochondrial ATP synthesis substantially decreased (Figure 5A,B). This inverse regulation resulted in a pronounced reduction of oxidative ATP generation accompanied by a compensatory increase in glycolytic ATP production.

Importantly, this metabolic shift was also observed in TGF-β-activated fibroblasts, indicating that NO-induced suppression of oxidative phosphorylation and enhancement of glycolysis occurs independently of the fibroblasts’ activation state (Figure 5).

### 3.5. ATP Rate Index and Total ATP upon DETA/NO Treatment in Both Untreated and TGF-β-Activated Fibroblasts

**Figure 6:**
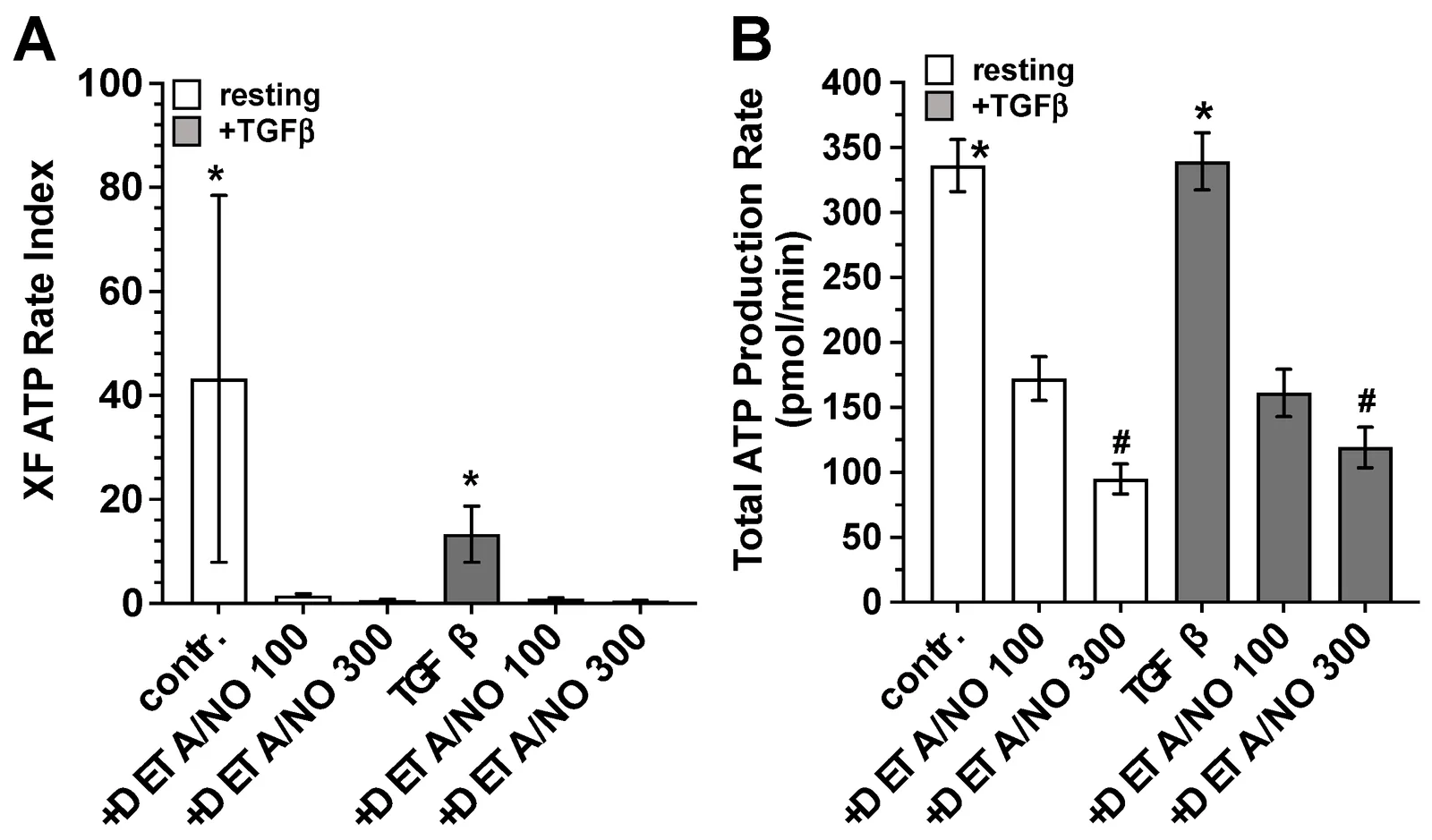
**ATP Rate Index and total ATP production upon DETA/NO treatment in untreated and TGF-β-activated fibroblasts.** Untreated (white bars) or TGF-β-activated (2 ng/mL; gray bars) primary human skin fibroblast cultures were maintained for 72 h in the presence or absence of the NO-donor DETA/NO (100 or 300 µM). Using the Seahorse XF ATP Rate Assay, mitochondrial and glycolytic ATP synthesis rates were recorded in parallel. (**A**), ATP Rate Index, reflecting the relative balance between mitochondrial and glycolytic ATP production; (**B**), Total ATP production rate (pmol/min). Values represent the mean ± S.D. of five individual experiments (five independent biological replicates; *n* = 5). *, *p* < 0.05 as compared to the respective DETA/NO-treated cultures; ^#^, *p* < 0.05 as compared to the respective cultures incubated with 100 µM DETA/NO.

Consequently, the ATP Rate Index, reflecting the relative balance between mitochondrial and glycolytic ATP production, was significantly reduced after DETA/NO treatment across all experimental groups (Figure 6A). Under control conditions, the index indicated a strong dominance of mitochondrial ATP production, whereas following NO exposure, a shift toward glycolysis as the dominant ATP-producing pathway was evident.

Additionally, total ATP production rates, measured in pmol/min, were assessed in untreated and TGF-β-activated fibroblasts under control conditions and following treatment with 100 µM and 300 µM DETA/NO (Figure 6B). In untreated fibroblasts, total ATP production was highest under control conditions and decreased significantly after exposure to DETA/NO in a dose-dependent manner. Treatment with 100 µM DETA/NO caused a moderate reduction, while 300 µM DETA/NO resulted in a more pronounced decrease in ATP synthesis. Similarly, TGF-β-activated fibroblasts showed a comparable pattern. Total ATP production under control conditions was high and significantly diminished upon treatment with both concentrations of DETA/NO, again demonstrating a dose-dependent effect. These results indicate that nitric oxide impairs overall ATP production regardless of fibroblast activation status, with higher DETA/NO concentrations causing stronger suppression of cellular energy metabolism (Figure 6).

### 3.6. NO Inhibits Myofibroblast Differentiation Independently of cGMP Signaling

**Figure 7:**
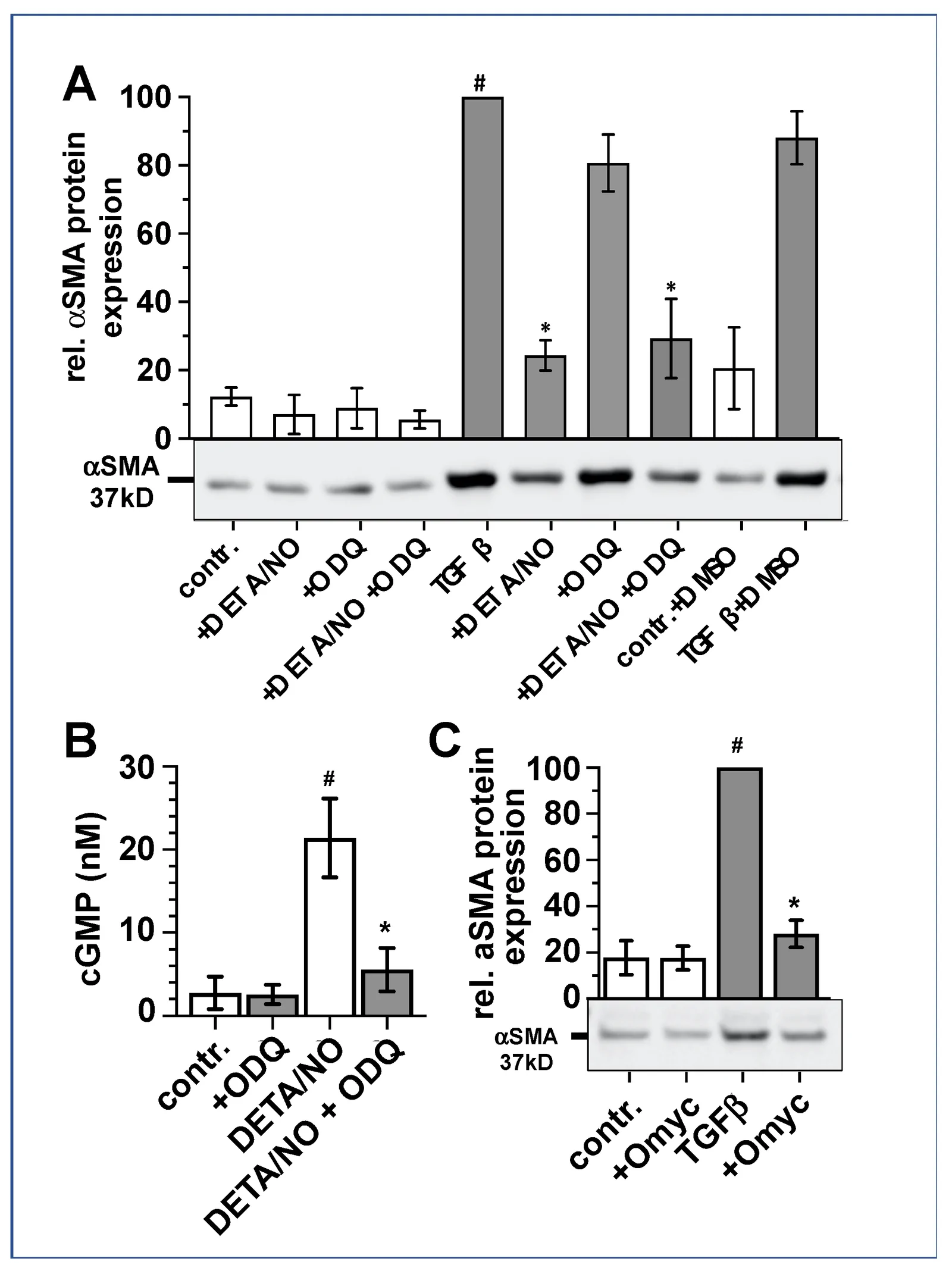
**Impact of sGC/cGMP signaling and mitochondrial ATP synthase inhibition on TGF-β-induced α-SMA protein expression.** (**A**), Analysis and densitometric quantification of α-SMA protein expression in untreated primary human dermal fibroblasts (white bars) and TGF-β-treated fibroblasts (gray bars) in the absence or presence of the NO donor DETA/NO (300 µM), the soluble guanylate cyclase (sGC) inhibitor ODQ (30 µM), or the respective combinations, and DMSO (2%) vehicle controls. Bars represent the mean ± SD of four (*n* = 4) individual experiments. ^#^, *p* < 0.05 as compared to the untreated control (contr.); *, *p* < 0.05 as compared to the corresponding TGF-β-treated group (TGFβ). (**B**), Determination of intracellular cGMP levels in NO-responsive RFL-6 reporter cells following treatment with DETA/NO (300 µM for 60 min) in the absence or presence of ODQ (30 µM; gray bars). Bars represent the mean ± SD of four individual experiments (four independent biological replicates; *n* = 4). ^#^, *p* < 0.05 as compared to the untreated control (contr.); *, *p* < 0.05 as compared to the corresponding DETA/NO-treated group (DETA/NO). (**C**), Representative Western blot analysis and densitometric quantification of α-SMA protein expression in untreated (white bars) as well as TGF-β-activated fibroblasts (gray bars) in the absence or presence of the mitochondrial ATP synthase inhibitor oligomycin C (+Omyc). Bars represent the mean ± SD of four individual experiments (four independent biological replicates; *n* = 4). ^#^, *p* < 0.05 as compared to the untreated control (contr.); *, *p* < 0.05 as compared to the corresponding TGF-β-treated group (TGFβ).

As shown in Figure 7A, treatment with the NO donor DETA/NO (300 µM), as expected, significantly attenuated TGF-β-induced α-SMA upregulation. Inhibition of the classical NO receptor, soluble guanylate cyclase, by 30 µM ODQ alone had no significant effect on basal or TGF-β-induced α-SMA expression. Importantly, co-treatment with ODQ failed to reverse the inhibitory effect of DETA/NO on α-SMA expression, indicating that the anti-fibrotic effects of NO occur largely independently of canonical sGC/cGMP signaling. DMSO control treatment did not affect α-SMA protein levels, excluding solvent-related effects.

To verify the biological activity and functional efficacy of ODQ, cGMP formation was analyzed in NO-responsive RFL-6 reporter cells (Figure 7B). DETA/NO treatment induced a robust increase in intracellular cGMP levels, whereas co-treatment with 30 µM ODQ significantly suppressed NO-induced cGMP accumulation. These findings confirm that ODQ efficiently inhibited sGC-dependent cGMP generation under the experimental conditions used.

To further examine whether impaired mitochondrial ATP production itself is sufficient to suppress fibroblast differentiation, cells were treated with the mitochondrial ATP synthase inhibitor oligomycin (Figure 7C). Similar to DETA/NO, oligomycin significantly reduced TGF-β-induced α-SMA protein expression. Notably, both interventions are characterized by impaired mitochondrial ATP production accompanied by compensatory glycolytic activation. These findings suggest that enhanced glycolysis alone is insufficient to maintain myofibroblast differentiation when overall cellular ATP availability is compromised.

## 4. Discussion

The present study identifies cellular energy availability as an important determinant of TGF-β-induced myofibroblast differentiation and reveals nitric oxide as a potent and reversible modulator of this metabolic requirement. Previous studies have established that fibroblast activation is accompanied by substantial metabolic reprogramming, with increased glycolytic activity being consistently associated with the fibrogenic phenotype [59,60]. Enhanced glycolysis has also been linked to the increased proliferative capacity of fibroblasts derived from hypertrophic scars [61], whereas inhibition of glycolytic metabolism reduces TGF-β-induced fibronectin and α-SMA expression and attenuates myofibroblast differentiation [35,41,60]. Together with broader evidence linking metabolic adaptation to fibroblast activation [35,37,38], these observations have supported the concept that increased glycolytic flux provides an important metabolic requirement for TGF-β-driven myofibroblast differentiation [35,39,41]. Our findings extend this concept by demonstrating that enhanced glycolytic ATP production alone is insufficient to sustain myofibroblast differentiation when mitochondrial ATP production and overall cellular energy availability are compromised.

This metabolic response is particularly relevant in the context of the established effects of NO on mitochondrial energy metabolism. Beyond its classical inflammatory and vascular functions, NO is known to reversibly inhibit cytochrome c oxidase (complex IV) of the mitochondrial electron transport chain [62,63]. This interaction directly modulates mitochondrial respiration, oxidative phosphorylation, and consequently mitochondrial ATP production. In addition, NO can influence cellular metabolism through post-translational modification of metabolic enzymes, including S-nitrosylation [64]. Thus, NO provides a biologically relevant means of transiently interfering with cellular bioenergetics and offers an experimental approach to examine whether increased glycolytic activity can compensate for impaired mitochondrial ATP production during fibroblast differentiation.

Our results demonstrate that this compensation is incomplete. DETA/NO markedly suppressed mitochondrial respiration and mitochondrial ATP production while simultaneously increasing glycolytic activity and glycolytic ATP generation. Nevertheless, total ATP production remained substantially reduced, and TGF-β-induced myofibroblast differentiation was strongly attenuated. Importantly, both mitochondrial respiration and the accompanying metabolic shift largely recovered following removal of the NO donor, supporting a reversible bioenergetic modulation rather than persistent mitochondrial dysfunction. These observations indicate that the metabolic requirements of myofibroblast differentiation cannot be defined by glycolytic activity alone but instead depend on the capacity of the cell to maintain adequate overall ATP production.

Additionally, NO has emerged as a context-dependent regulator of fibrotic remodeling, exhibiting both pro- and antifibrotic properties depending on its cellular source, concentration, redox environment, and disease stage. Evidence from multiple organ systems indicates that the biological impact of NO in fibrosis cannot be generalized but instead reflects isoform-specific and microenvironment-dependent signaling effects. In several experimental models, increased expression of inducible nitric oxide synthase (iNOS) has been associated with enhanced fibrotic progression. In the bleomycin-induced mouse model of pulmonary fibrosis, iNOS deficiency significantly attenuated collagen deposition and reduced fibrotic remodeling, indicating a pro-fibrotic contribution of iNOS-derived NO [65]. Similarly, pharmacologic inhibition of iNOS reduced lung fibrosis severity in murine models [66]. Mechanistically, excessive iNOS activity has been linked to peroxynitrite formation, oxidative stress, and sustained inflammatory signaling, all of which can amplify TGF-β-driven fibrotic responses [67]. Comparable findings have been reported in renal fibrosis. In models of chronic kidney injury, increased iNOS expression correlates with matrix accumulation and tubulointerstitial fibrosis [68,69]. These studies suggest that sustained inflammatory NO production may promote fibrogenesis under conditions of chronic tissue injury. In contrast, endothelial nitric oxide synthase (eNOS)-derived NO frequently exerts protective effects. In experimental pulmonary fibrosis, eNOS-deficient mice develop more severe fibrotic changes compared to wild-type controls, suggesting a protective vascular and antifibrotic role of basal NO signaling [70,71]. Similarly, in liver fibrosis models, eNOS deficiency aggravates collagen deposition and portal hypertension [72]. Direct antifibrotic effects of exogenous NO have also been documented. In cultured dermal fibroblasts, NO donors reduce collagen synthesis and attenuate profibrotic activation via cGMP-dependent mechanisms [73,74]. This duality underscores the importance of interpreting NO effects within specific experimental frameworks and supports a nuanced view in which nitric oxide may either exacerbate or restrain fibrotic remodeling depending on the biological context.

In the present study, we demonstrate that exogenous nitric oxide (NO), delivered via the donor DETA/NO, exerts a potent antifibrotic effect on human dermal fibroblasts by suppressing TGF-β-induced myofibroblast differentiation. This was evidenced by a concentration-dependent reduction in α-SMA mRNA and protein expression levels, as well as diminished stress fiber formation observed by immunocytochemistry. The concentrations of exogenously applied NO required to achieve significantly reduced differentiation of activated human skin fibroblasts fall within the physiological range, as indicated by published studies. Previous work [75] demonstrated that DETA/NO releases NO spontaneously according to predictable first-order kinetics, with a half-life of approximately 7.7 h, thereby ensuring a sustained and relatively constant NO supply over several hours. Based on first-order kinetic equations (t_1_/_2_ = ln2/k and c = c_0_ × e^−kt^), 1 mM DETA/NO yields approximately 2.7 µM NO per minute. Other estimates suggest that a steady-state NO concentration of around 4–5 µM is present in the immediate vicinity of a cell monolayer capable of enzymatic NO production [76]. Taken together, these calculations and experimental findings indicate that the 300 µM DETA/NO levels may generate NO concentrations that overlap with those reported in highly activated cellular microenvironments.

Mechanistically, metabolic profiling using Seahorse XF analysis demonstrated that DETA/NO acutely and reversibly suppresses mitochondrial oxidative phosphorylation in primary human dermal fibroblasts. Basal respiration, ATP-linked respiration, and maximal respiratory capacity were significantly reduced in a concentration-dependent manner after 8 h of NO exposure. Importantly, these effects were not associated with irreversible mitochondrial dysfunction, as respiratory parameters substantially recovered following a 24 h washout period. In parallel, DETA/NO induced a marked transient increase in basal glycolysis and glycolytic capacity, consistent with a compensatory metabolic adaptation to impaired mitochondrial ATP generation. These glycolytic alterations also normalized after removal of the NO donor, further supporting a reversible metabolic reprogramming rather than persistent cellular injury.

The present functional analyses do not establish whether this reversible bioenergetic response is accompanied by alterations in mitochondrial abundance, morphology, biogenesis, or turnover. Although the rapid suppression and subsequent recovery of mitochondrial respiration are consistent with reversible functional regulation of oxidative phosphorylation, additional adaptive mitochondrial responses cannot be excluded. Mitochondrial dynamics and quality-control mechanisms, including mitophagy and autophagy, as well as changes in mitochondrial biogenesis or expression of oxidative phosphorylation-related proteins, could contribute to cellular adaptation and metabolic recovery. Elucidating whether such processes contribute to the coupling between cellular energy availability and myofibroblast differentiation will require dedicated structural and molecular analyses and represents an important direction for future studies.

Detailed ATP rate analyses further revealed that under basal conditions, fibroblasts rely predominantly on mitochondrial oxidative phosphorylation, which accounted for approximately 95% of total ATP production. Exposure to DETA/NO caused a profound shift in cellular energy metabolism characterized by a strong suppression of mitochondrial ATP synthesis to approximately 35% of baseline values, whereas glycolytic ATP production increased to approximately 70% in both untreated and TGF-β-activated fibroblasts. Despite this compensatory glycolytic response, total ATP production remained markedly reduced by approximately 70%, indicating that enhanced glycolysis alone was insufficient to compensate for impaired mitochondrial ATP generation. Notably, these effects were largely independent of the TGF-β-induced activation status of the fibroblasts and fully reversible following removal of NO.

The present findings therefore challenge the prevailing glycolysis-centered paradigm of myofibroblast differentiation. Particularly noteworthy is the observation that DETA/NO markedly increased glycolytic ATP production while simultaneously suppressing α-SMA expression and myofibroblast differentiation. These findings indicate that enhanced glycolytic activity alone is insufficient to sustain the fibrogenic phenotype. Rather, maintenance of cellular ATP homeostasis appears to be more critical for differentiation than glycolytic flux per se. Previous studies proposed that enhanced glycolytic flux and broader metabolic reprogramming contribute to fibroblast activation, myofibroblast differentiation, and fibrotic remodeling [35,37,38]. In contrast, our data demonstrate that a substantial increase in glycolysis can occur concomitantly with profound suppression of TGF-β-induced myofibroblast differentiation. DETA/NO markedly inhibited α-SMA expression despite a pronounced increase in glycolytic ATP production, while simultaneously suppressing mitochondrial respiration and reducing total cellular ATP availability. These observations suggest that glycolytic activation per se is insufficient to sustain myofibroblast differentiation when mitochondrial ATP production and overall cellular energy homeostasis are compromised. Consequently, total ATP availability and intact mitochondrial bioenergetic function may represent more critical determinants of differentiation capacity than glycolytic rate alone. This interpretation is further supported by studies demonstrating that TGF-β-induced fibroblast differentiation is associated with increased mitochondrial content, increased mitochondrial respiration, and broader energetic remodeling [29,77].

Importantly, inhibition of soluble guanylate cyclase by ODQ failed to reverse the NO-mediated suppression of α-SMA expression, although ODQ efficiently blocked NO-induced cGMP generation in RFL-6 reporter cells. These findings argue against a predominant role of canonical NO-sGC-cGMP signaling in mediating the observed anti-differentiative effects of NO in dermal fibroblasts and instead support a cGMP-independent mechanism linked to metabolic modulation and mitochondrial respiratory inhibition. This interpretation is consistent with the well-established ability of NO to reversibly interfere with mitochondrial respiration at the level of cytochrome c oxidase (complex IV) in competition with oxygen [62].

To further examine whether impaired mitochondrial ATP production itself is sufficient to suppress fibroblast differentiation, oligomycin was used as a functional bioenergetic control. Similar to DETA/NO, inhibition of mitochondrial ATP synthase by oligomycin significantly reduced TGF-β-induced α-SMA expression. This finding strongly supports the concept that adequate cellular ATP production appears to be required for full myofibroblast differentiation. Of course, the mechanistic actions of oligomycin and NO are not directly equivalent. While NO predominantly and reversibly interferes with mitochondrial respiration at the level of cytochrome c oxidase (complex IV), oligomycin directly inhibits the mitochondrial ATP synthase (complex V) by binding to the Fo/c-ring region and blocking proton translocation [78]. Nevertheless, the bioenergetic effects of oligomycin closely mirrored those observed following NO exposure. Importantly, inhibition of ATP synthase by oligomycin is known to trigger a compensatory increase in glycolytic ATP production. Thus, despite increased glycolytic activity, myofibroblast differentiation remained strongly suppressed. The observation that two mechanistically distinct interventions converge on a comparable metabolic and phenotypic outcome further supports the concept that glycolytic activation alone is insufficient to maintain fibroblast differentiation when overall ATP availability is reduced. Instead, our findings suggest that adequate mitochondrial ATP production and overall energy availability may represent critical determinants of the differentiation process. Thus, oligomycin cannot be regarded as a mechanistically identical surrogate for NO signaling but rather as a functional control demonstrating that disruption of mitochondrial ATP generation itself is sufficient to impair fibroblast differentiation. Together, these findings support the concept that mitochondrial bioenergetics function as a metabolic checkpoint controlling fibroblast activation and differentiation.

An important implication of our findings is that NO-mediated metabolic modulation is likely to be most effective during the early phases of fibroblast activation and myofibroblast differentiation. Since our experiments primarily demonstrate inhibition of TGF-β-induced differentiation rather than reversal of an established myofibroblast phenotype, the therapeutic potential of NO-based interventions may reside predominantly in the prevention of excessive scar formation rather than in the treatment of mature fibrotic lesions. Consequently, NO-mediated metabolic targeting may be particularly attractive as a prophylactic strategy during wound healing and early tissue remodeling, when fibroblast activation remains plastic and potentially reversible.

A limitation of the present study is that all experiments were performed under conventional cell culture oxygen conditions. Oxygen tension can modulate fibroblast differentiation and TGF-β signaling and is particularly relevant to NO-mediated regulation of mitochondrial respiration because the interaction of NO with cytochrome c oxidase is oxygen-dependent. Consequently, the magnitude of the metabolic and anti-differentiative effects observed here may differ under lower tissue oxygen tensions. Whether physiological or pathophysiological oxygen gradients modify the NO-dependent coupling between mitochondrial ATP production and myofibroblast differentiation therefore represents an important question for future studies. Another potential limitation of the present study may be the use of culture medium supplemented with 10% FCS, which may contain variable amounts of endogenous TGF-β. Although previous studies have demonstrated that TGF-β present in FCS predominantly exists in a latent form [79], its potential activation during cell culture cannot be excluded. Nevertheless, under our experimental conditions, untreated control fibroblasts exhibited only low basal α-SMA expression, whereas the addition of exogenous TGF-β1 resulted in a pronounced induction of α-SMA. Thus, although potential batch-dependent variations in serum composition may influence cellular responsiveness, our results clearly demonstrate a TGF-β1-dependent induction of myofibroblast differentiation under the experimental conditions employed.

Collectively, our data identify nitric oxide as a potent and reversible regulator of fibroblast metabolism and differentiation. More broadly, our findings challenge the concept that enhanced glycolysis represents a sufficient metabolic driver of myofibroblast differentiation. Instead, they suggest that preservation of cellular ATP homeostasis is a critical prerequisite for acquisition and maintenance of the myofibroblast phenotype. By transiently suppressing mitochondrial oxidative phosphorylation and cellular ATP availability independently of canonical cGMP signaling, NO effectively attenuates TGF-β-driven myofibroblast differentiation. These findings suggest that targeted and reversible modulation of mitochondrial bioenergetics may represent a promising therapeutic strategy for the prevention or treatment of pathological fibrosis and hypertrophic scar formation.

## Data Availability

Upon individual request, all data underlying this project can be made available to the interested parties. The datasets used and/or analyzed during the current study are available from the corresponding author on reasonable request.
